# Supplementary material for: Icatibant Acts as a Balanced Ligand of MRGPRX2 in Human Skin Mast Cells
Source: Biomolecules. 2025 Aug 25;15(9):1224. doi: 10.3390/biom15091224 (PMC12466958; doi:10.3390/biom15091224)
Supplement: Supplementary file 1 [file biomolecules-15-01224-s001.zip › biomolecules-3765342-supplementary.pdf]

biomolecules

MDPI

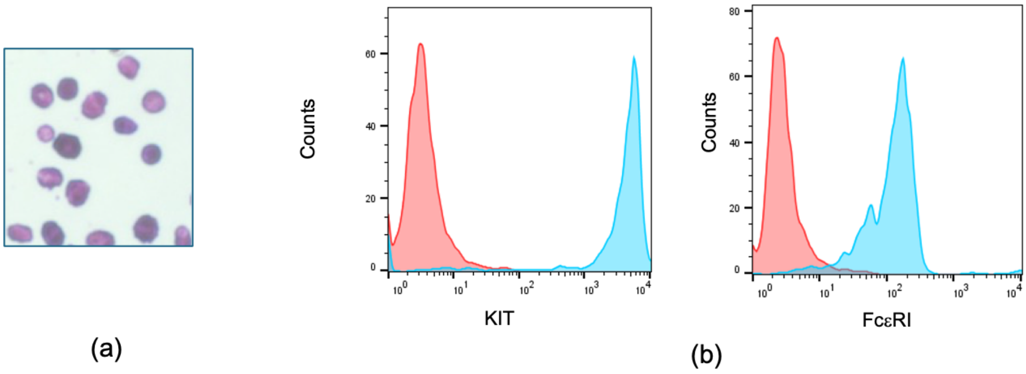


**Figure S1.** Phenotypic validation of isolated human skin MCs.(**a**) Acidic toluidine blue staining of MCs isolated from human foreskin tissue. (**b**) Flow cytometry histograms showing surface expression of KIT (CD117) and FcεRI (IgE receptor). Red: isotype control, blue: receptor-specific antibody.

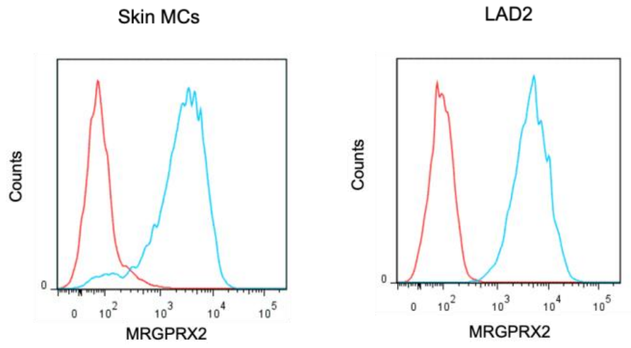


**Figure S2.** Representative histograms of MRGPRX2 surface expression in skin MCs and LAD2 cells. Red: isotype control, blue: MRGPRX2.

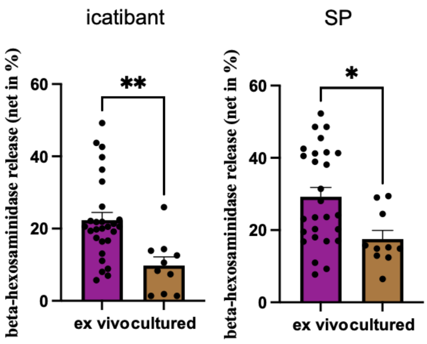


**Figure S3.** Comparison of icatibant- and SP-induced degranulation responses in ex vivo (purple) and cultured (brown) skin MCs. Degranulation responses of cultured skin MCs compared to ex vivo skin MCs, under the same stimulation conditions as in Figure. 1. Each dot represents an individual skin MC preparation/culture. The data are the mean ± SEM of $n$ = 10–28. * $p < 0.05$, ** $p < 0.01$.

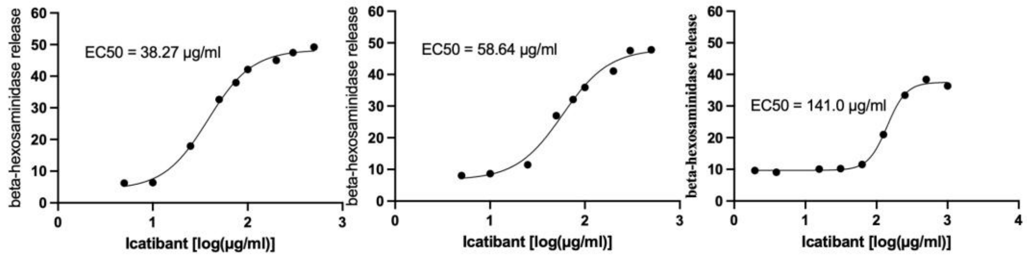


**Figure S4.** Dose response curves of degranulation responses triggered by icatibant in skin MCs. Each curve was generated with MCs from a distinct donor pool.

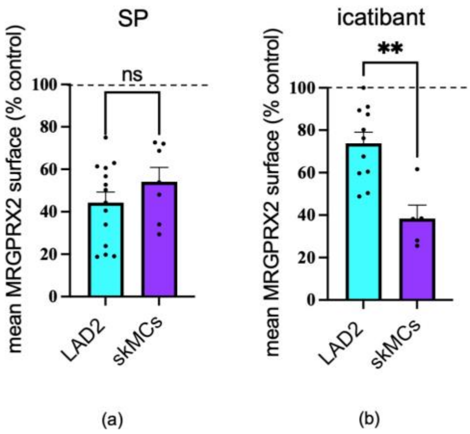


**Figure S5.** Comparative MRGPRX2 internalization in LAD2 cells and skin MCs after 1-hour stimulation with agonists. skin MCs and LAD2 cells were stimulated for 1 hour by (**a**) SP or (**b**) icatibant. Unstimulated cells served as control. MRGPRX2 surface expression was measured and normalized to control. The data are as in main Figure 4 but shown in a different way. ns: not significant, ** $p < 0.01$. Dashed line: no internalization.

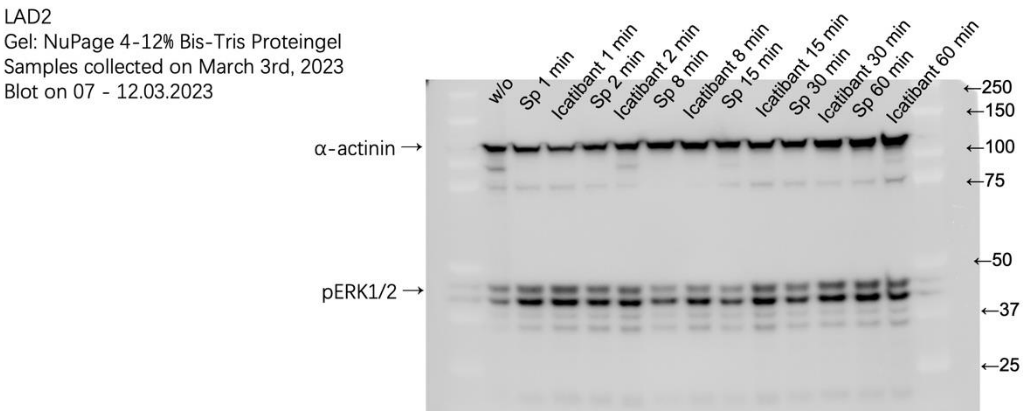


Densitometry:

| | w/o | SP 1min | icatibant 1min | SP 2min | icatibant 2min | SP 8min | icatibant 8min | SP 15min | icatibant 15min | SP 30min | icatibant 30min | SP 60min | icatibant 60min |
|---|---|---|---|---|---|---|---|---|---|---|---|---|---|
| α-actinin | 10013 | 12175 | 10361 | 12765 | 14330 | 15347 | 14668 | 14122 | 13721 | 13291 | 16011 | 17004 | 18666 |
| pERK1 | 2327 | 5214 | 7300 | 7068 | 8466 | 6462 | 8200 | 6243 | 11143 | 8218 | 9623 | 8484 | 7631 |
| pERK2 | 1976 | 7274 | 10223 | 10138 | 11149 | 8567 | 10481 | 8119 | 12981 | 10170 | 12834 | 11549 | 9631 |

LAD2
Gel: NuPage 4-12% Bis-Tris Proteingel
Samples collected on March 3rd, 2023
Blot on 07 - 12.03.2023

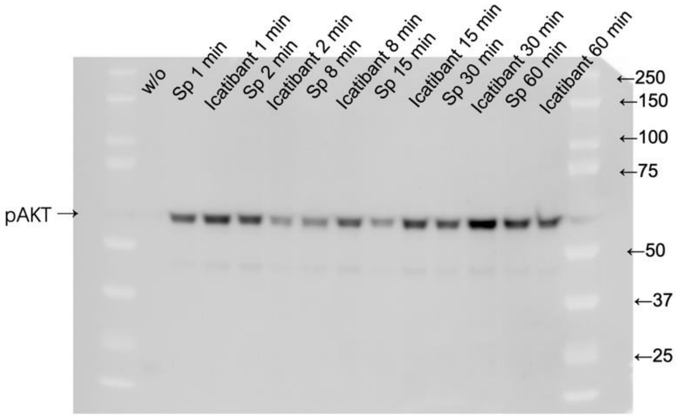


Densitometry:

| w/o | SP | icatibant | SP | icatibant | SP | icatibant | SP | icatibant | SP | icatibant | SP | icatibant |
|---|---|---|---|---|---|---|---|---|---|---|---|---|
| | 1min | | 2min | | 8min | | 15min | | 30min | | 60min | |
| 397 | 7376 | 9347 | 7952 | 3991 | 5365 | 7187 | 4048 | 8606 | 7272 | 12534 | 9300 | 8350 |

LAD2
Gel: NuPage 4-12% Bis-Tris Proteingel
Samples collected on March 3rd, 2023
Blot on 07 - 12.03.2023

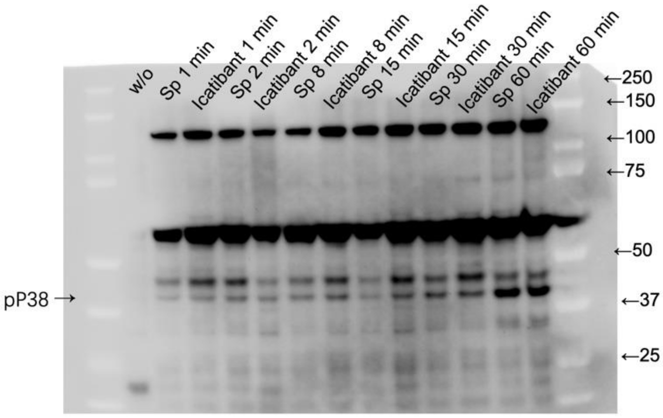


Densitometry:

| w/o | SP | icatibant | SP | icatibant | SP | icatibant | SP | icatibant | SP | icatibant | SP | icatibant |
|---|---|---|---|---|---|---|---|---|---|---|---|---|
| | 1min | | 2min | | 8min | | 15min | | 30min | | 60min | |
| 131 | 1640 | 2576 | 2917 | 1996 | 2956 | 3521 | 1400 | 2251 | 2586 | 4060 | 10882 | 10555 |

LAD2
Gel: NuPage 4-12% Bis-Tris Proteingel
Samples collected on March 3rd, 2023
Blot on 07 - 12.03.2023

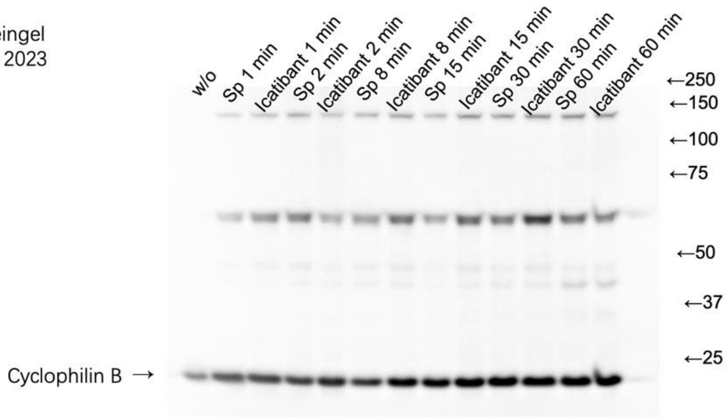


Densitometry:

| w/o | SP | icatibant | SP | icatibant | SP | icatibant | SP | icatibant | SP | icatibant | SP | icatibant |
|---|---|---|---|---|---|---|---|---|---|---|---|---|
| | 1min | | 2min | | 8min | | 15min | | 30min | | 60min | |
| 3844 | 9188 | 10329 | 9596 | 11218 | 9326 | 12129 | 12413 | 13879 | 14796 | 14407 | 14499 | 13897 |

FsMc-31(13,04,2023)
Gel: NuPage 4-12% Bis-Tris Proteingel
Samples collected on April 14th, 2023
Blot on 18-20,04,2023

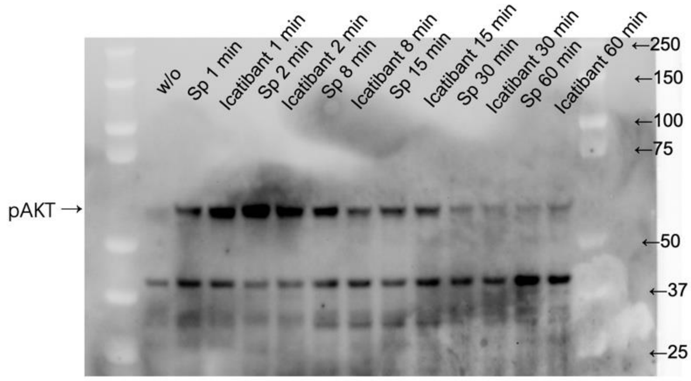


Densitometry:

| w/o | SP 1min | icatibant 1min | SP 2min | icatibant 2min | SP 8min | icatibant 8min | SP 15min | icatibant 15min | SP 30min | icatibant 30min | SP 60min | icatibant 60min |
|---|---|---|---|---|---|---|---|---|---|---|---|---|
| 3813 | 8721 | 12584 | 14353 | 9418 | 12174 | 5397 | 8344 | 7341 | 3137 | 3388 | 2533 | 4092 |

FsMc-31(13,04,2023)
Gel: NuPage 4-12% Bis-Tris Proteingel
Samples collected on April 14th, 2023
Blot on 18-20,04,2023

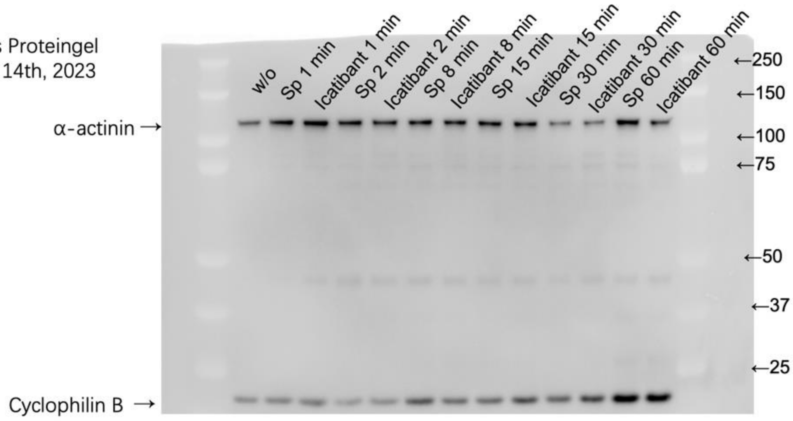


Densitometry:

| | w/o | SP 1min | icatibant 1min | SP 2min | icatibant 2min | SP 8min | icatibant 8min | SP 15min | icatibant 15min | SP 30min | icatibant 30min | SP 60min | icatibant 60min |
|---|---|---|---|---|---|---|---|---|---|---|---|---|---|
| α-actinin | 4797 | 8585 | 10462 | 8917 | 8249 | 8620 | 8913 | 9241 | 7934 | 4023 | 4313 | 9884 | 6112 |
| Cyclophilin B | 5784 | 7635 | 6872 | 3589 | 4747 | 8648 | 7803 | 8433 | 10952 | 9103 | 11640 | 11813 | 9191 |

FsMc-27(30,03,2023)
Gel: NuPage 4-12% Bis-Tris Proteingel
Samples collected on March 31rd, 2023
Blot on 04-06.04.2023

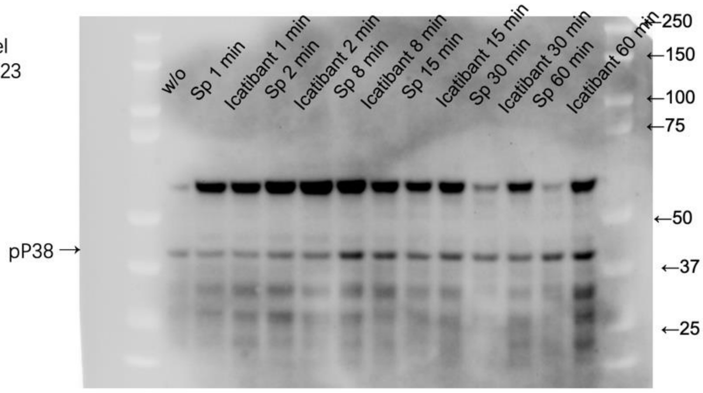


Densitometry:

| w/o | SP 1min | icatibant 1min | SP 2min | icatibant 2min | SP 8min | icatibant 8min | SP 15min | icatibant 15min | SP 30min | icatibant 30min | SP 60min | icatibant 60min |
|---|---|---|---|---|---|---|---|---|---|---|---|---|
| 3228 | 4133 | 4406 | 5343 | 4706 | 8519 | 7704 | 5447 | 7272 | 5906 | 5037 | 7410 | 9461 |

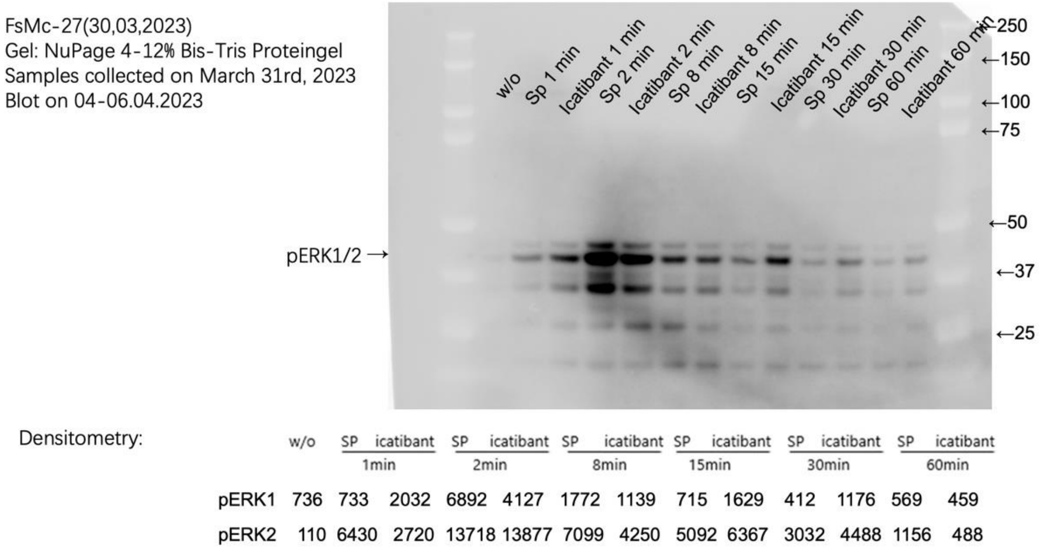


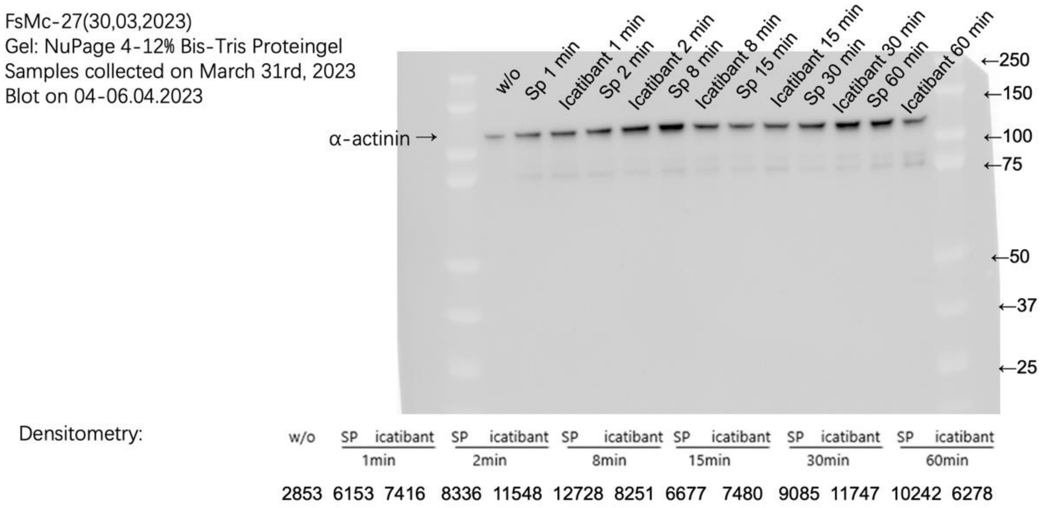


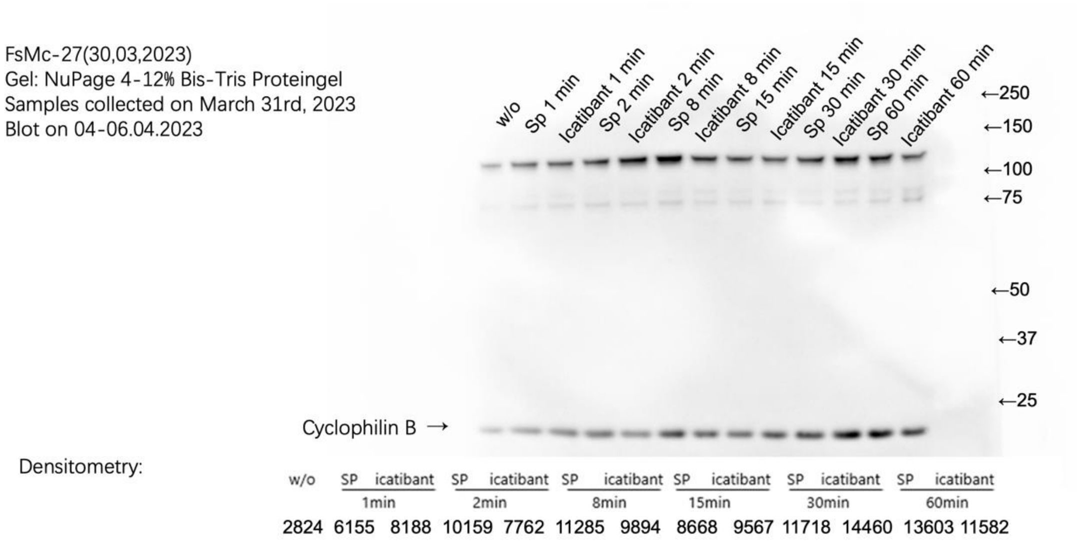


**Figure S6.** Original Western blot image of Figure 2.
